# Supplementary material for: Deciphering direct transcriptional effects of epigenetic compounds through large-scale new RNA profiling
Source: Nat Commun. 2025 Jul 18;16:6629. doi: 10.1038/s41467-025-61769-z (PMC12274403; doi:10.1038/s41467-025-61769-z)
Supplement: Supplementary file 1 — Supplementary Information [file 41467_2025_61769_MOESM1_ESM.pdf]

Supplementary Information

Deciphering direct transcriptional effects of epigenetic compounds through large-scale new RNA profiling

Leonard Hartmanis[1], Daniel Ramsköld[1], Gert-Jan Hendriks[1], Per Johnsson[1], Gustav Hallén[1], Ran Ma[2], Anton J.M. Larsson[1], Salomé Hahne[1], Christoph Ziegenhain[1], Johan Hartman[2,3] and Rickard Sandberg[1,*]

[1]Department of Cell and Molecular Biology, Karolinska Institutet, Stockholm, Sweden

[2]Department of Oncology-Pathology, Karolinska Institutet, Stockholm, Sweden

[3]Department of Clinical Pathology and Cancer Diagnostics, Karolinska University Hospital, Stockholm, Sweden

*correspondence to: R.S. (Rickard.Sandberg@ki.se)

Supplementary Figures 1-10.

Supplementary Figure 1

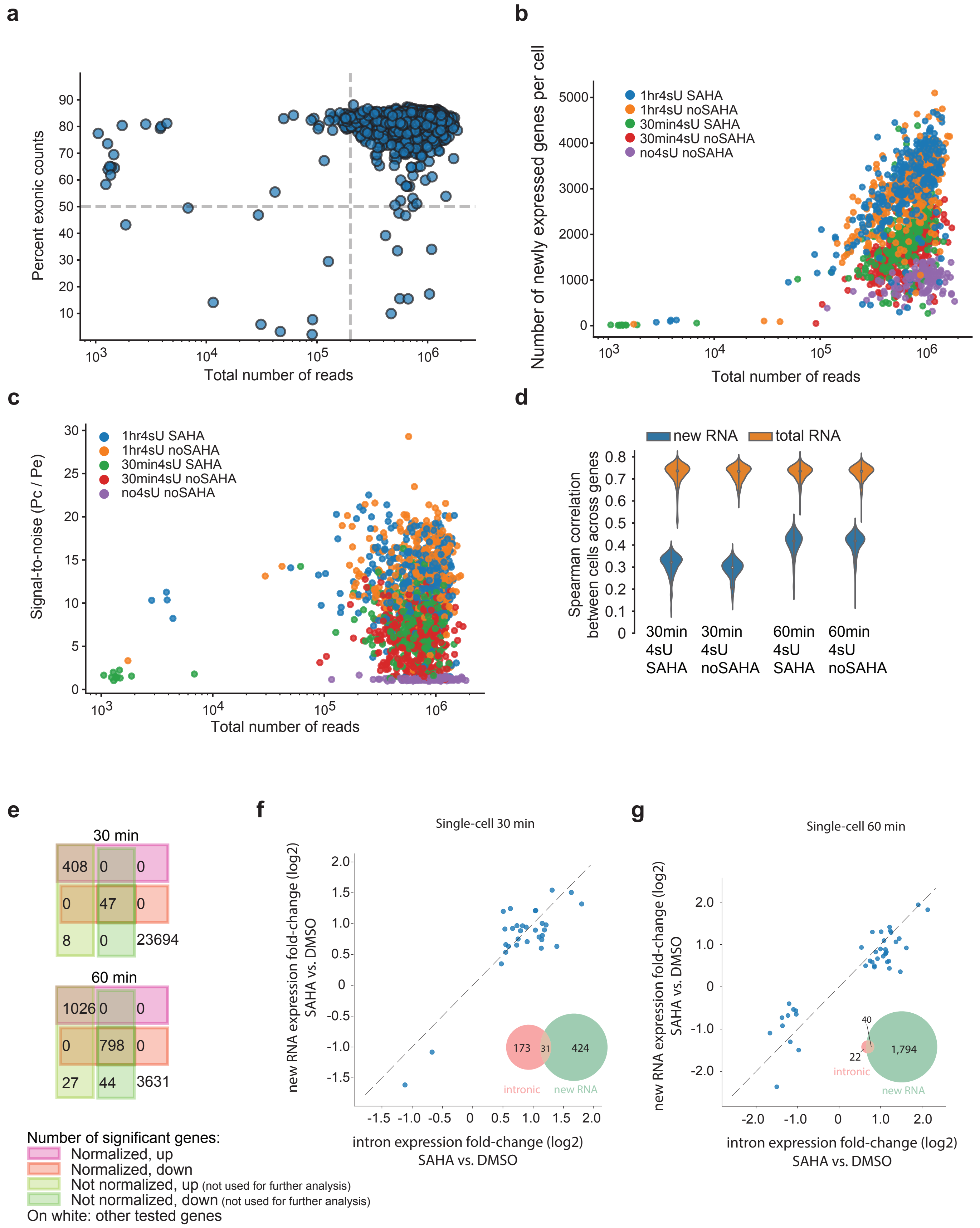

Supplementary Figure 1. Quality statistics of NASC-seq2 sequencing data.

(**a**) Scatter plot showing the percent of sequencing reads mapping to exons and the total number of reads, per cell. Cells in the upper right quadrant are selected for further analyses. (**b**) Scatter plot showing the number of newly expressed genes detected per cell as against the total number of reads, per cell and colored according to 4sU labeling time. (**c**) Scatter plot showing the signal to noise ratio (p_c / p_e) against the total number of reads, per cell and colored according to 4sU labeling time. (b-c) The residual background of new RNA observations for samples not subject to 4sU likely comes from genetic polymorphisms and instable inference for low-expressed genes (as shown in Supplementary Figure 3). (**d**) Cell to cell gene expression profile correlations for new and total RNA. (**e**) Comparing the number of differentially expressed genes from DESeq2 on either normalized or non-normalized reads. The consistency in the results show that the DESeq2 normalization does not create false positives. (**f**) Correlation of differentially expressed genes detected for cells treated with SAHA for 30 minutes compared to untreated control cells, using expression levels quantified from new RNA molecules versus expression levels quantified total RNA and reads mapping introns (upper). Venn diagram showing the overlap of differentially expressed genes detected in new molecules compared to total intron data. (**g**) Same as in (f) but for 60-minute SAHA treatment. Source data are provided as a Source Data file.

Supplementary Figure 2

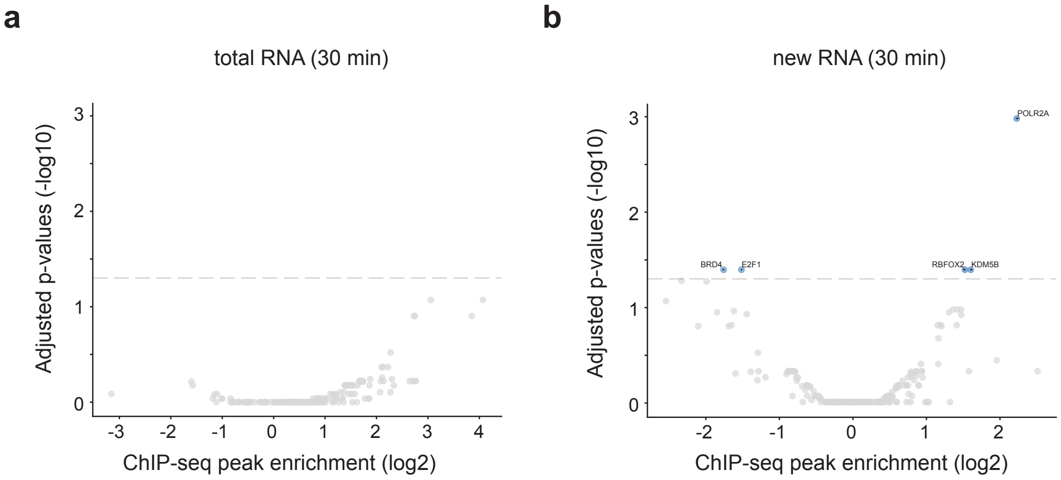

Supplementary Figure 2. Enrichment of ChIP-seq bound factors at genes identified after 30-minute SAHA treatment in K562 cells.

(**a-b**) Volcano plot showing enrichment of ENCODE ChIP-seq peaks in promoters of up- and down-regulated genes after 30 minutes SAHA treatment for total RNA (a) and new RNA (b). The up-regulated genes were compared against the down-regulated genes. Enrichments were evaluated using Fisher's exact test (two-sided), with the resulting P values adjusted using Benjamini-Hochberg procedure. Source data are provided as a Source Data file.

Supplementary Figure 3

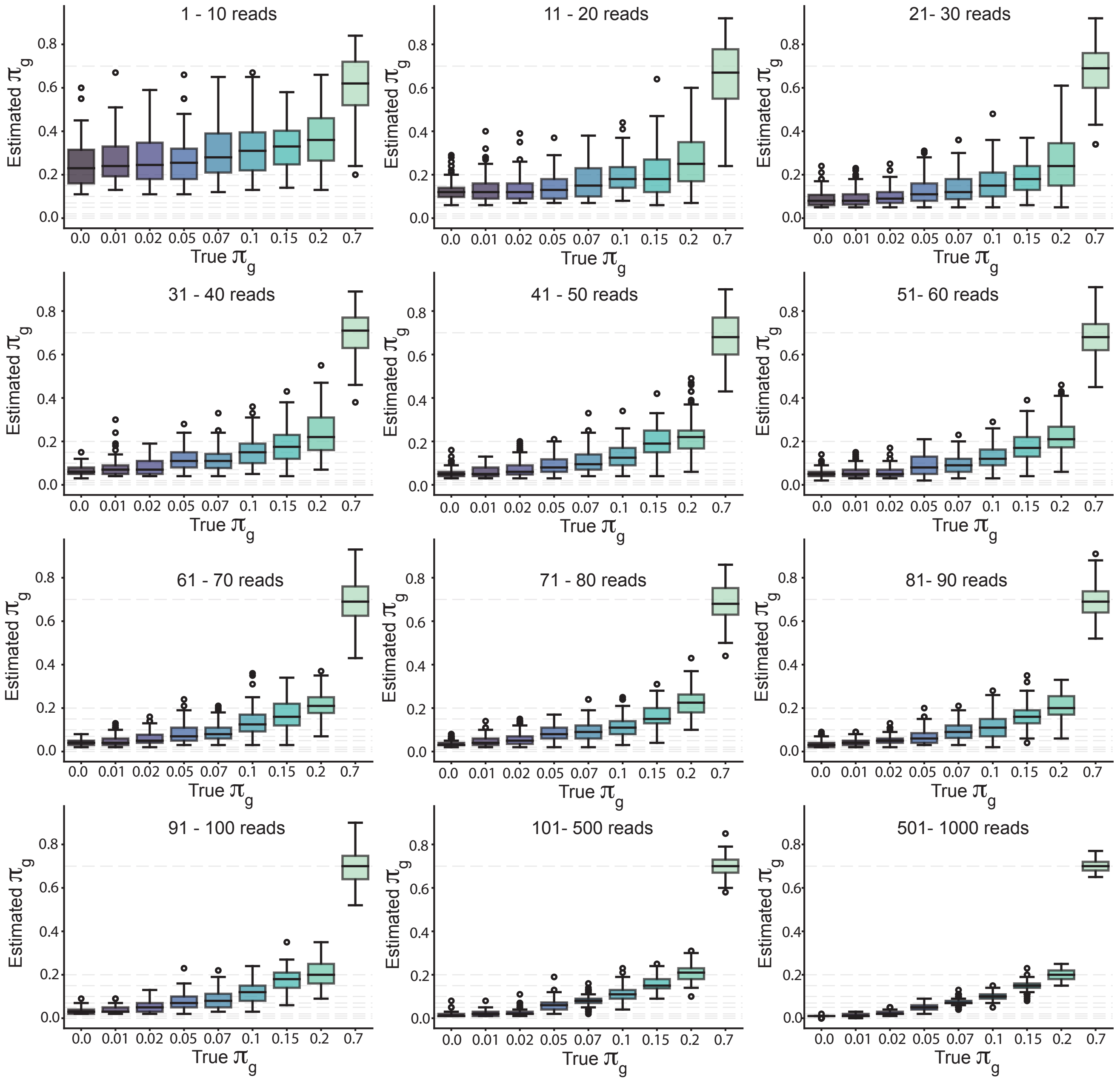

Supplementary Figure 3. Benchmarking new RNA inference using simulations.

Boxplots showing the results from MCMC based π_g inference (fraction new RNA) on simulated data. Data was simulated from conditions with a similar signal-to-noise ratio as detected in our mini-bulk experiment (p_c 0,03 and p_e 0,003), 100 data-points was simulated for each read depth range and true π_g value. Horizontal dashed lines mark the true value. Source data are provided as a Source Data file.

Supplementary Figure 4

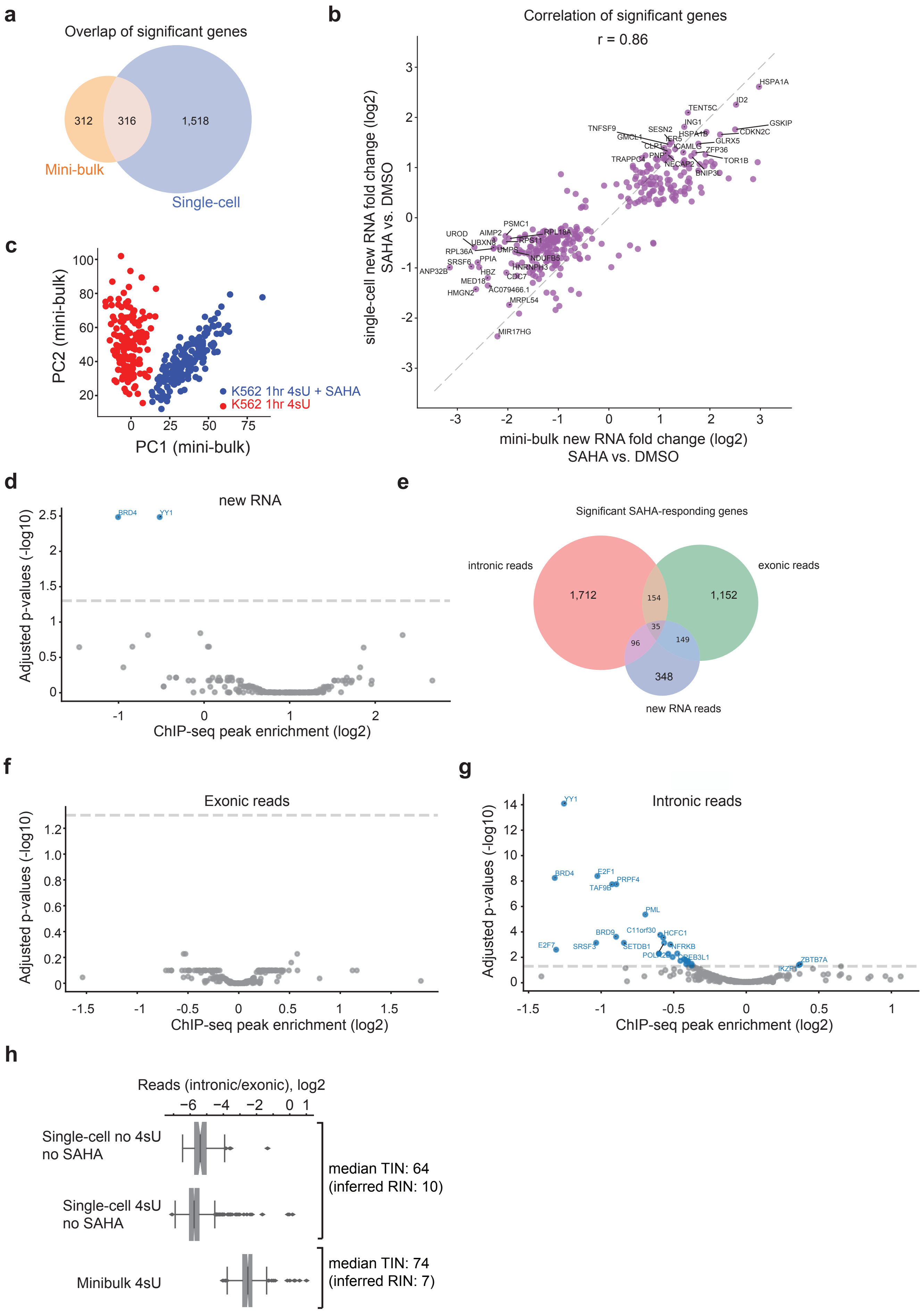

Supplementary Figure 4. Comparing the effect of SAHA on new RNA profiles in mini-bulk and NASC-seq2 experiments on K562 cells. (**a**) Venn diagram of new RNA differentially expressed genes in mini-bulk and NASC-seq2 data. (**b**) Scatter plot of new RNA fold changes (SAHA treated against DMSO controls) for significant genes detected in the NASC-seq2 (y-axis) and mini-bulk experiments (x-axis), together with the Spearman correlation. (**c**) Projection of NASC-seq2 data onto the PCA loadings computed from the mini-bulk experiment, showing that similar new RNA direct effects were identified. (**d**) Volcano plot showing enrichment of ENCODE K562 ChIP-seq peaks of specific factors in the genes with significant change in new RNA profiles upon 1-hour SAHA treatment (upregulated vs downregulated) in the mini-bulk experiment on K562 cells. Enrichments were evaluated using Fisher's exact test (two-sided), with the resulting P values adjusted using Benjamini-Hochberg procedure. (**e**) Overlap of significant differentially expressed genes found when considering total RNA reads mapping to introns, total RNA mapping to exons and new RNA mapping to exons (**f**) Enrichment of transcription factor binding at promoters in upregulated genes compared to downregulated genes detected in total RNA with expression levels quantified from reads mapping to exons (i.e., not consider 4sU labeling). (**g**) Same as in (f) but for differentially expressed genes with gene expression quantified from reads mapping to introns. (f-g) Enrichments were evaluated using Fisher's exact test (two-sided), with the resulting P values adjusted using Benjamini-Hochberg procedure. (**h**) Boxplots showing per-sample ratios of reads mapping to intronic versus exonic regions. Notches show bootstrapped 95% confidence intervals. We calculated transcript integrity (TIN) numbers, a computational measure of 3’ bias that translate into approximate RIN numbers[33], which revealed lower TIN/RIN numbers for the single-cell data. Source data are provided as a Source Data file.

Supplementary Figure 5

a

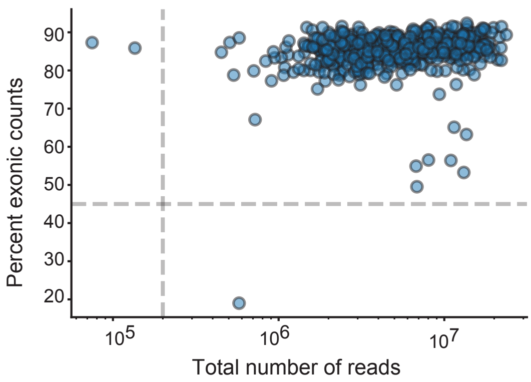

b

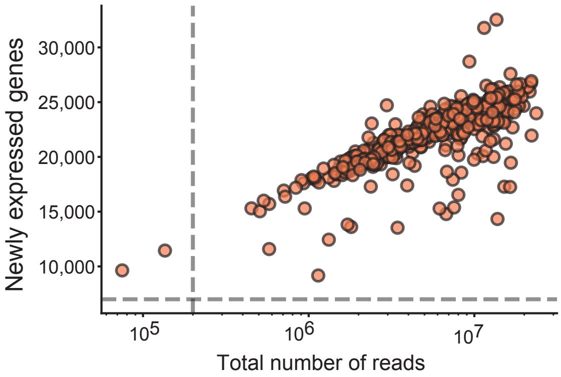

c

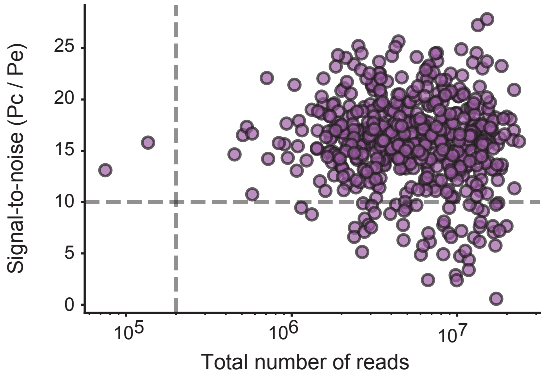

Supplementary Figure 5. Quality statistics of sequence data in mini-bulk screen of epigenetic compounds. (**a**) Scatter plot showing the percent of sequencing reads mapping to exons and the total number of reads, per sample. Samples in the upper right quadrant are selected for further analyses. (**b**) Scatter plot showing the number of newly expressed genes detected per cell as against the total number of reads, per sample. (**c**) Scatter plot showing the signal to noise ratio (p_c / p_e) against the total number of reads, per sample. Source data are provided as a Source Data file.

Supplementary Figure 6

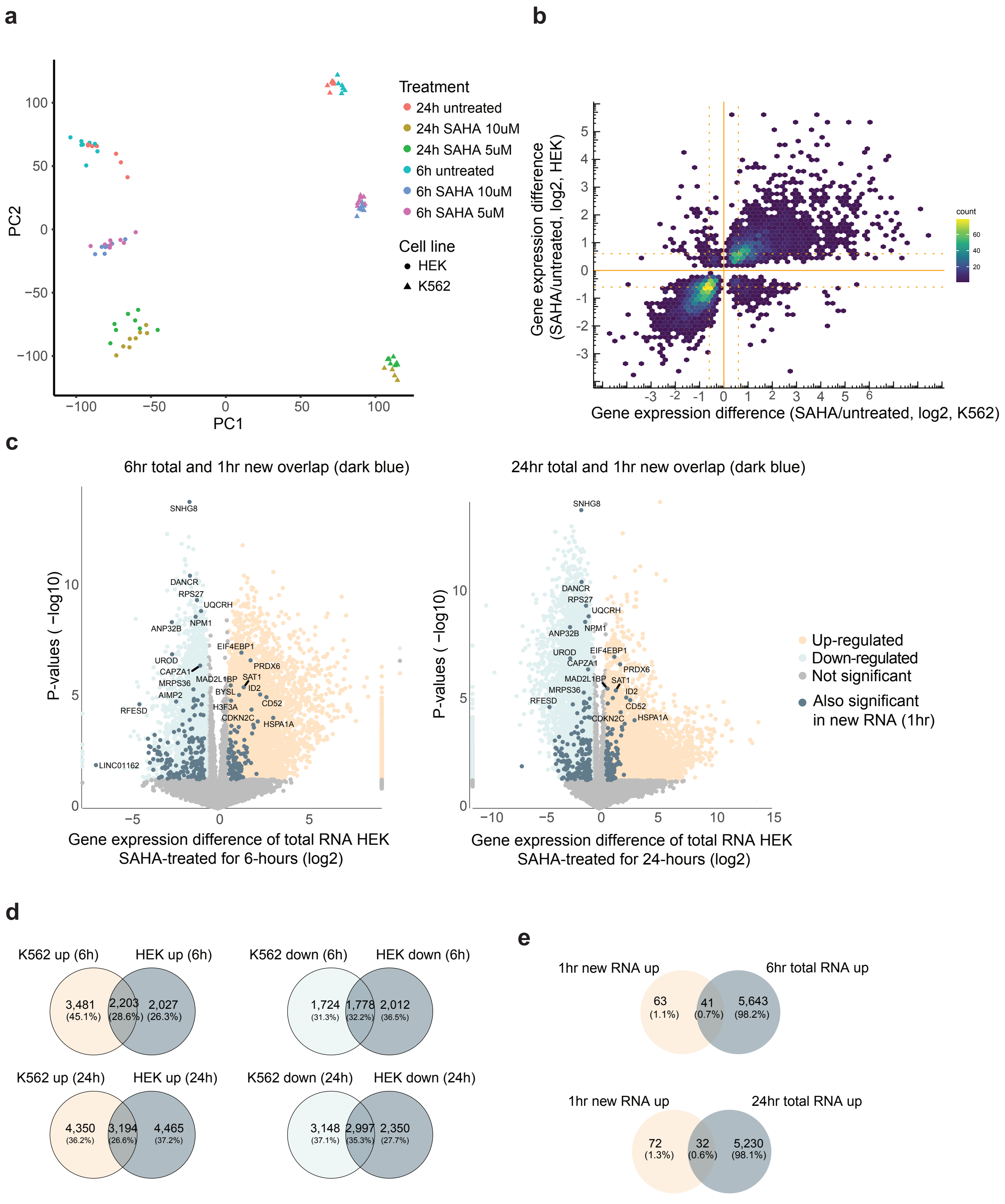

Supplementary Figure 6. Transcriptional effects of longer-term SAHA treatment in K562 and HEK293FT cells. (**a**) Principal component analysis of total RNA profiles after SAHA treatment for 6 or 24 hours, per cell line and SAHA dose, showing that cell line and treatment time are the strongest determinants of the transcriptional response. (**b**) Correlation of differentially expressed genes detected in the two cell lines after SAHA-treatment for 6 hours at 5 µM. (**c**) Volcano plots of differentially expressed cells after 24 hours (left) and 6 hours (right) SAHA treatment. (**d**) Venn diagrams showing the overlap of differentially expressed upregulated (left column) and downregulated (right column) genes between the two cell lines after 6 hours (top row) and 24 hours (bottom row) treatment (10 µM). (**e**) Venn diagrams showing the overlap of differentially expressed genes detected in new RNA after 1 hours SAHA treatment compared to significant genes in total RNA after 6 hours (top) and 24 hours (bottom) treatments, both using 10 µM SAHA treatments. Source data are provided as a Source Data file.

Supplementary Figure 7

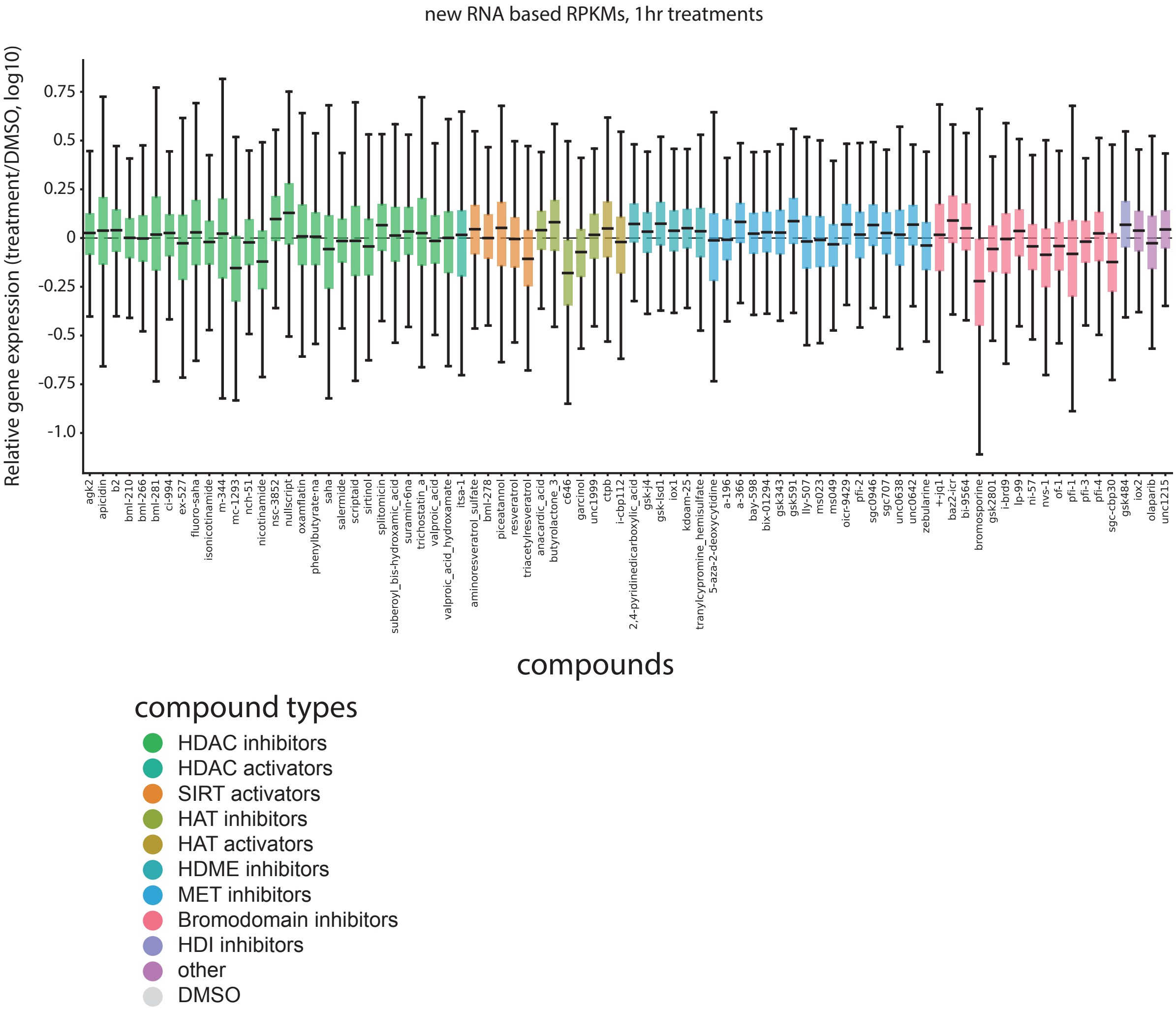

Supplementary Figure 7. Overall changes in new RNA levels per compound. Boxplots showing the distributions of the gene-wise fractions of mean new RNA based RPKM per treatment compared DMSO treated controls, for all genes with detected new RNA expression. Source data are provided as a Source Data file.

Supplementary Figure 8

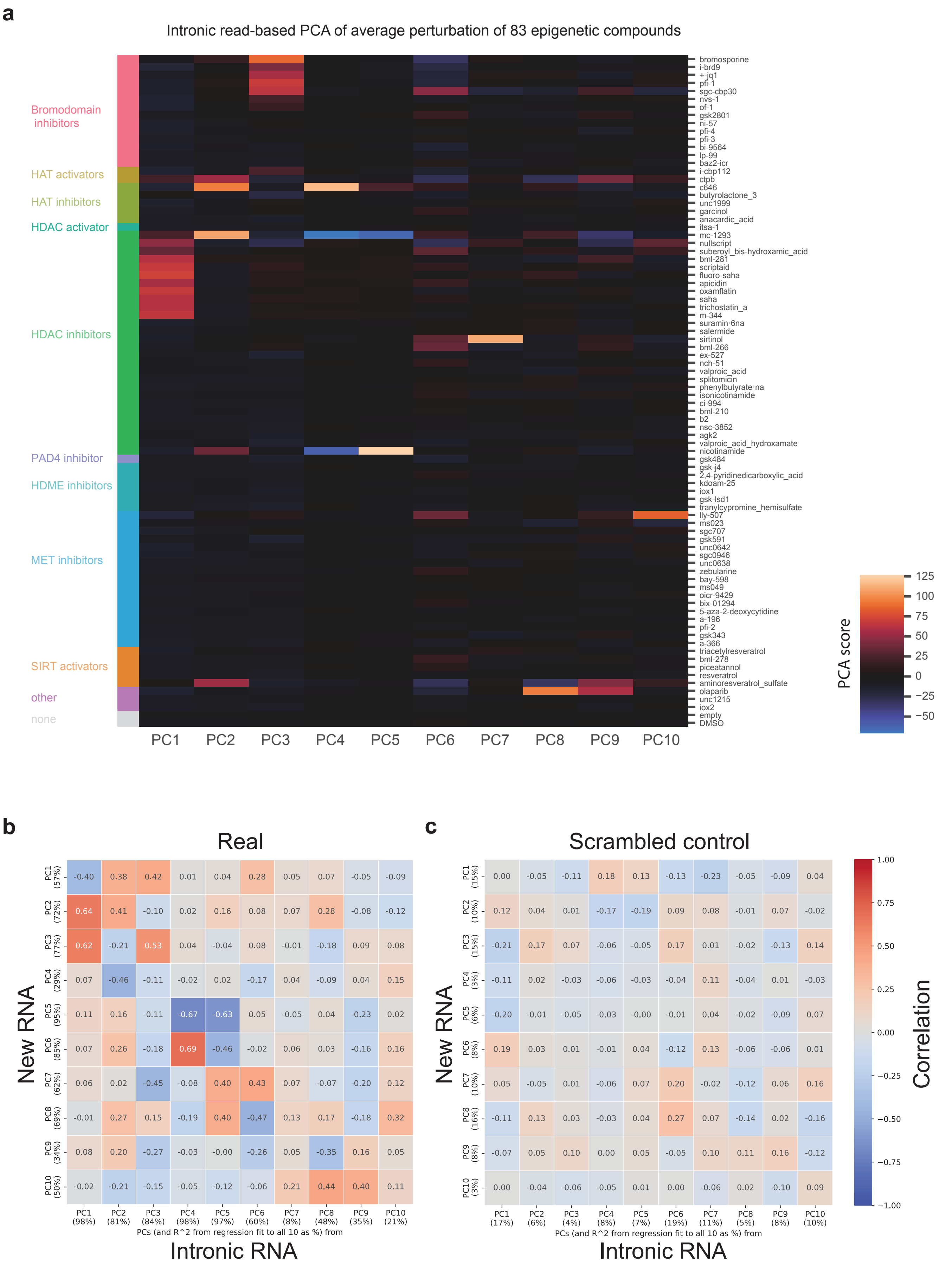

Supplementary Figure 8. PCA analysis of intronic expression patterns after perturbations with 83 epigenetic compounds. (**a**) PCA scores computed from the union of differentially expressed genes detected in total intron data for mini-bulk samples treated with the drug panel of 83 epigenetically active compounds. (**b**) Correlations between the principal components in (a) and those in Figure 4c for new RNA. R^2 values (explained variance) from linear regression of one principal component against the 10 principal components in the other set are shown in parentheses at the edges. These values illustrate how many intron-data principal components are simply linear combinations of new RNA principal components, rather than truly differing. (**c**) Like (b), but with genes shuffled, demonstrating the sizes of random correlations between principal components, reaching no higher than $r = 0.27$ for a fit to one principal component and $R^2 = 19\%$ for a fit to 10 principal components, in contrast to the larger numbers in (b). Source data are provided as a Source Data file.

Supplementary Figure 9

a

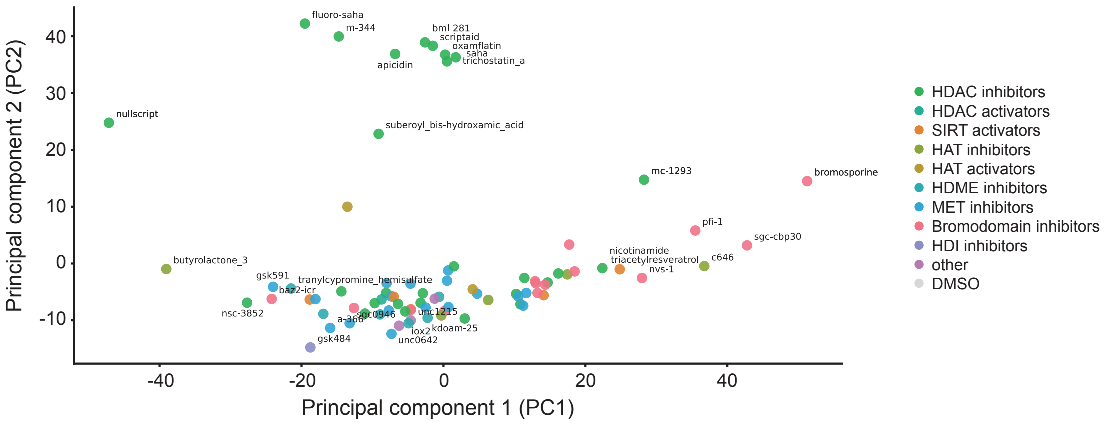

b

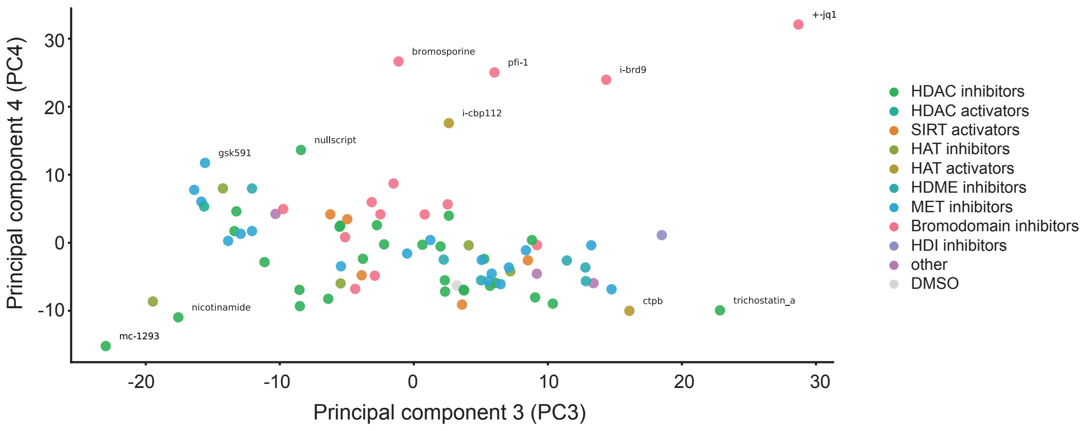

Supplementary Figure 9. PCA analysis of new RNA effects across all 83 epigenetic compounds. (**a**) Scatter plot of principal component 1 vs principal component 2 (**b**) and principal component 4 vs principal component 3. Each compound is colored coded based on overall compound type. Source data are provided as a Source Data file.

Supplementary Figure 10

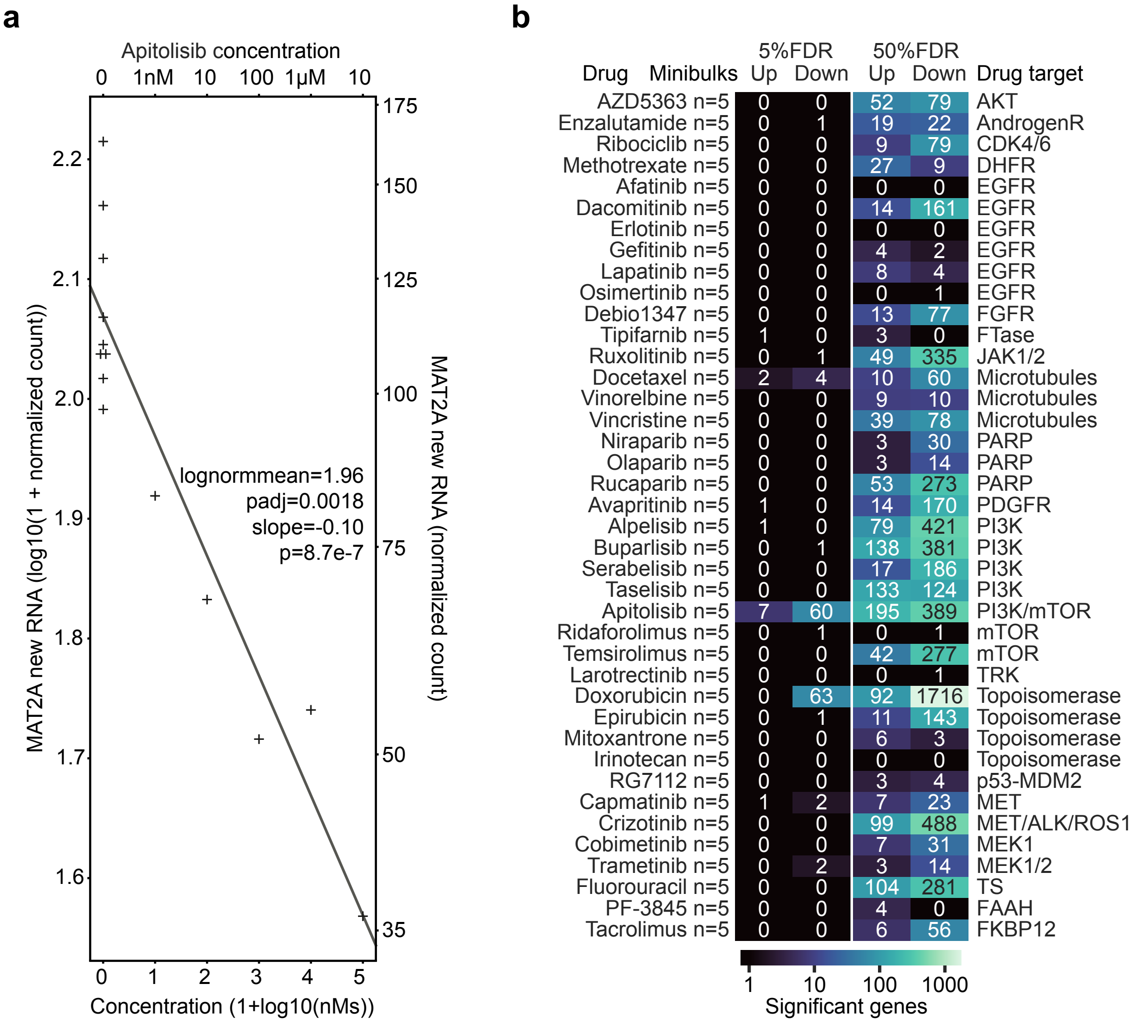

Supplementary Figure 10. Compound screen in MCF7 cells. (**a**) An example of the test employed: least squares linear regression of five concentrations plus nine negative controls that are the same for the comparison with every drug, with a Wald test of a t-distribution (scipy.stats.linregress). Due to the log-uniformly distributed concentrations and the presence of zeros, we have log10(1+conc) transformed the x-axis and ln(1+count) transformed the y axis prior to regression, as the highest concentration alone would otherwise drive the result. The line shows the fit from the same python function as the p-values come from. The gene is considered downregulated in this treatment since the slope is negative. (**b**) A list of compounds and the number of significant genes at a 5% and 50% FDR cutoff. The 50% cutoff is later used for gene ontology since that analysis method handles noisy input without necessarily producing noisy output. Source data are provided as a Source Data file.
