# Supplementary material for: Deciphering direct transcriptional effects of epigenetic compounds through large-scale new RNA profiling
Source: Nat Commun. 2025 Jul 18;16:6629. doi: 10.1038/s41467-025-61769-z (PMC12274403; doi:10.1038/s41467-025-61769-z)
Supplement: Supplementary file 2 — Description of Additional Supplementary Files [file 41467_2025_61769_MOESM2_ESM.pdf]

Description of Additional Supplementary Files

File Name: Supplementary Data 1
Description: Differential expression after SAHA treatment, single-cell experiment.
Analysis of differential expression is presented for SAHA-treated K562 cells after 30 or 60 minutes. The analyses based on new RNA and total RNA (new and pre-existing) are presented in separate tabs. Differential expression was performed using DESeq2, comparing SAHA-treated samples for each time point against treatment time-matched DMSO controls, showing both nominal p-values and adjusted p-values (Bonferroni).

File Name: Supplementary Data 2
Description: Differential expression based on new RNA after treatments with 83 compounds.
Analysis of differential expression after treatment with each compound in K562 cells is shown in separate tabs. To test for differentially expressed genes, we used a t-test between a gene's expression in the compound-treated samples and 10 randomly sampled DMSO controls. Only compounds with three replicates were used, and the DMSO control samples were independently sampled for each test. To account for the low number of replicates, we utilized a variance adjustment approach, in which we first estimated a gene's expected variance based on its expression level through either linear or lowess regression. If a gene had a lower variance than the regression predicted, we used the predicted value in the t-test instead. After significance testing, we adjusted the p-values for multiple hypothesis testing using a two-stage FDR correction.

File Name: Supplementary Data 3
Description: Regressions of gene expression values against compound doses.
Compound panel in MCF7 cells, with nine untreated controls and five treated samples at concentrations of 1 nM, 10 nM, 100 nM, 1 μM, and 10 μM. Statistical tests were performed using the Python function scipy.stats.linregress. The results for each compound are shown on separate tabs/sheets.
